# Transfer and Integration of Breast Milk Stem Cells to the Brain of Suckling Pups

**DOI:** 10.1038/s41598-018-32715-5

**Published:** 2018-09-24

**Authors:** Mehmet Şerif Aydın, Esra Nur Yiğit, Emre Vatandaşlar, Ender Erdoğan, Gürkan Öztürk

**Affiliations:** 1 Regenerative and Restorative Medicine Research Center, Istanbul Medipol University, Istanbul, 34810 Turkey; 2 Department of Histology and Embryology, Faculty of Medicine, Selcuk University, Konya, 42030 Turkey; 3 Department of Physiology, International School of Medicine, Istanbul Medipol University, Istanbul, 34810 Turkey

## Abstract

Beside its unique nutritional content breast milk also contains live cells from the mother. Fate of these cells in the offspring has not been adequately described. In this study, we aimed to detect and identify maternal cells in the suckling’s blood and the brain. Green fluorescent protein expressing transgenic female mice (GFP+) were used as foster mothers to breastfeed wildtype newborn pups. One week and two months after the birth, blood samples and brains of the sucklings were analyzed to detect presence of GFP+ cells by fluorescence activated cell sorting, polymerase chain reaction and immunohistochemistry on the brain sections and optically cleared brains. The tests confirmed that maternal cells were detectable in the blood and the brain of the pups and that they differentiated into both neuronal and glial cell types in the brain. This phenomenon represents breastfeeding – induced microchimerism in the brain with functional implications remain to be understood.

## Introduction

The breast milk is a unique secretion that contains different bioactive substances such as hormones, enzymes, immunoglobulins, growth factors, cytokines, anti-inflammatory agents and anti-microbial factors beside the nutritional content composed of proteins, carbohydrates, fats and vitamins^1^. It also harbors live cells of different types that vary in distribution significantly. While leukocytes constitute 13–70% of all breast milk cells (BMCs) under normal conditions, this rate may rise up to 94% in an infection^2^. Epithelial cells from the ducts of mammary glands are always among the normal cellular population^3^. Another group of cells in the breast milk is mammary gland stem cells (BSc) that provide the formation of new mammary tissue during lactogenesis^4^. While BSc are found in few numbers or inactive in a normal mammary gland, they actively regenerate the mammary gland with pregnancy and breastfeeding. They can differentiate into alveolar, ductal and myoepithelial cells of mammary tissue^5^. Indeed, an entirely new breast formation was achieved in BSc transplanted mice^6^. Rather curiously, beside BSc, the breast milk contains other types of stem cells that express embryonic markers like nestin, cytokeratin, OCT4, SOX2, NANOG, SSEA4 and TRA-1^4,7,8^. These cells were successfully differentiated into neurons, hepatocytes, pancreatic beta cells, osteoblasts, and adipocytes under *in vitro* conditions^4^.

The breast milk – born cells have been shown to survive the challenging conditions of gastrointestinal tract of an infant and pass to the intestinal wall^9^ and blood circulation that carries them to the liver^10^ and the spleen^11^. However, exact distribution of these cells in the body and their fate are largely unknown. Possibility of breast milk stem cells to differentiate and to get integrated into different tissues has been speculated but not conclusively proved^12^.

A first attempt to decipher transfer and potential integration of breastmilk-derived stem cells, along with immune cells, to the offspring was recently carried out by Hassiotou (now Kakulas) *et al*. with positive results showing integration and differentiation in various organs of the nursed offspring in a murine model^13^. Consistent with these earlier reports, in this study, we have shown that, breast milk stem cells pass to the pups, reach to the brain, settle there and differentiate into neuron and glial cells in mice.

## Results

### Detection of GFP+ Cells in the Bloodstream and Brain of Pups by Flow Cytometry

**Figure 1:**
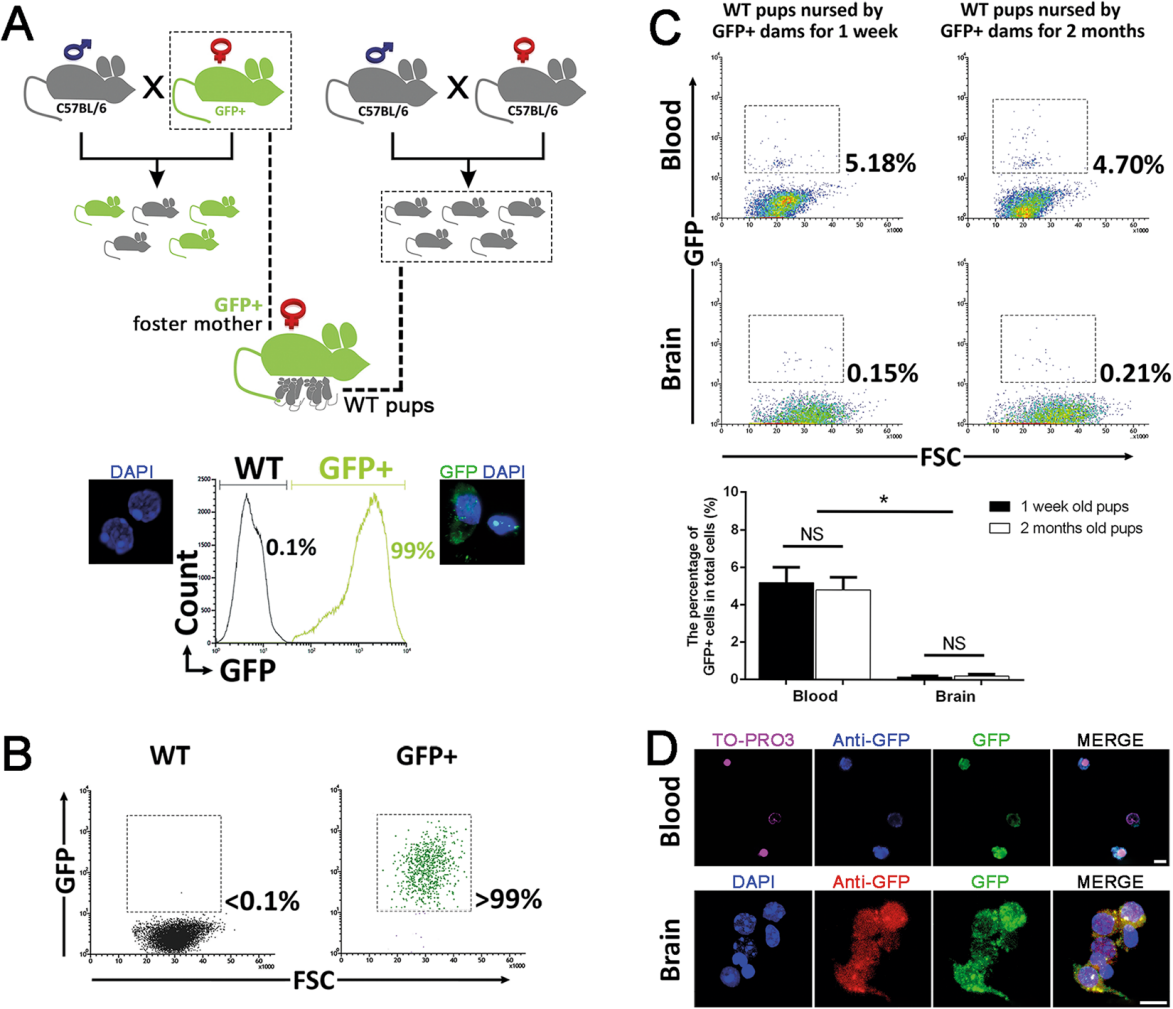
Experimental design and flow cytometric analyses. (**A**) Breeding and nursing diagram of WT and GFP+ mice. WT newborn pups were immediately delivered to GFP+ foster mothers to be breastfed. Flow cytometry plots present the confirmation of expected GFP expression in the brains of GFP+ and WT mice. (**B**) Brain samples of WT and GFP+ mice were analyzed with GFP/FSC plot to determine the threshold of GFP signal for further flow cytometry analysis. (**C**) GFP+ cells were detected in both blood and brain of pups by the end of first week (n = 6) and two months (n = 6) of nursing. Error bars in the percentage of GFP+ cells in total cells graphs are S.D. (**D**) GFP+ cells detected in the brain by flow cytometry were sorted to verify GFP signal microscopically (scale bars: 10 µm).

To detect the transfer of milk cells, we made GFP+ female mice breastfeed WT pups postpartum. Background noise and threshold of the GFP and anti-GFP signal was determined using positive (GFP+) and negative (WT) control cell suspensions prepared from freshly dissected brain tissues with isotype controls. The cells that had both GFP signal and anti-GFP staining were considered to be of breast milk origin. As expected, we found >99% of GFP+ cells in the positive control group while <0.1% in the negative control group (Fig. 1). We found GFP+ cells in the bloods of pups which were nursed by GFP+ dams for 1 week (5.18 ± 2.1%) and for 2 months (4.7 ± 1.6%). We also detected GFP+ cells in the brain tissue of pups which were nursed by GFP+ dams for 1 week (0.15 ± 0.1%) and for 2 months (0.21 ± 1%). There was no statistically significant difference in GFP+ cell numbers between 1 week and 2 months nursing periods (Supplementary Table S1). Cells identified as GFP+ by flow cytometry were sorted and examined by confocal microscope and GFP and anti-GFP signals were verified (Fig. 1).

### Characterization of GFP+ Cells in Brain by Immunohistochemistry

**Figure 2:**
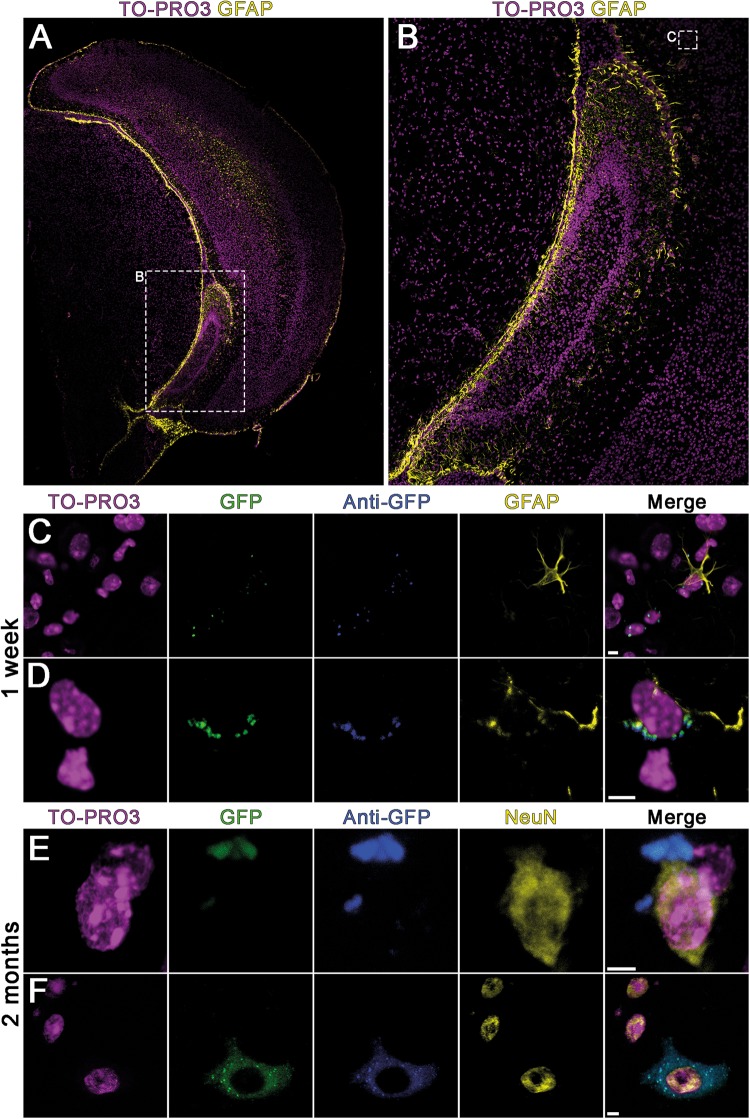
Immunohistochemically stained brain sections of sucklings nursed by GFP+ mothers. Breast milk borne maternal cells are randomly distributed in the pup’s brain. (**A**,**B**) Representative zoomed images of GFP+ cells in the hippocampal region of the brain. (**C**–**F**) Glial cell marker (anti-GFAP) and neuronal marker (anti-NeuN) were used to label different cell types, counterstained with nuclear dye TO-PRO3. GFP+ cells differentiated to neuronal and glial cells were detected after one week and two months of nursing (scale bars: 5 µm).

**Figure 3:**
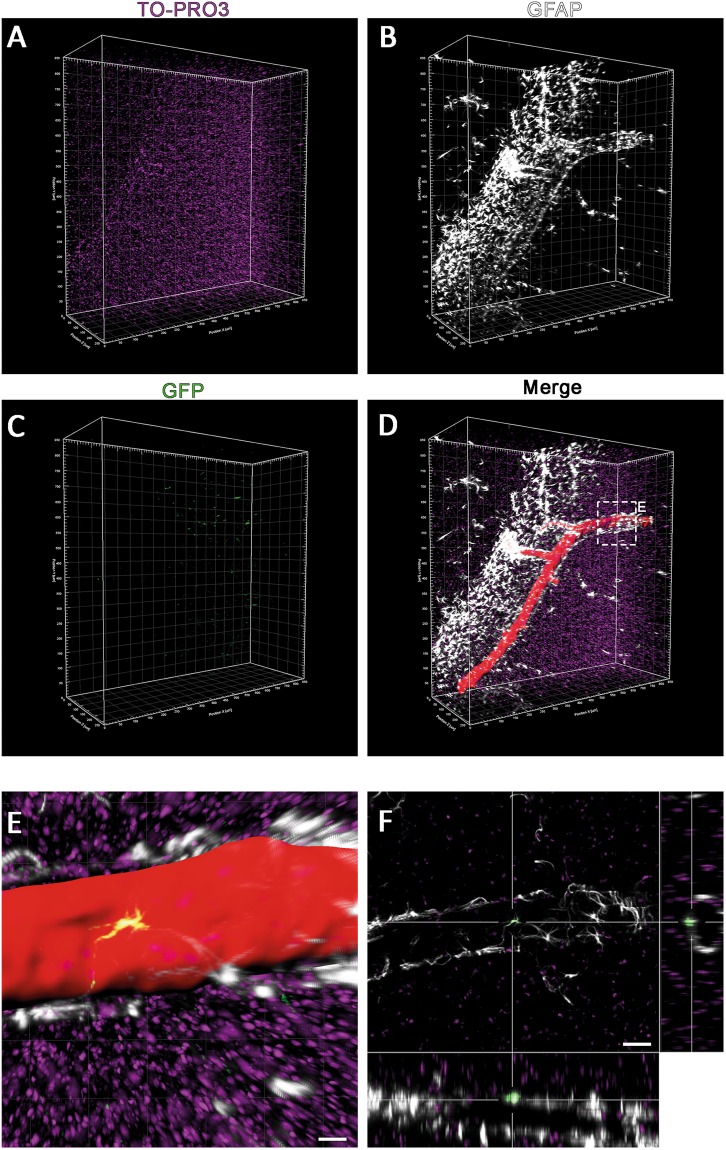
Tissue clearing method and immunohistochemical staining of sucklings brain. iDISCO method was used to determine the specific localization and 3-dimensional morphology of the GFP+ cells in the pup’s brain. GFP+ cells showed a random distribution like in sections. However, the morphology and localization of these cells were much better elucidated in cleared tissue. (**A**–**D**) GFAP+ and GFP+ cells can be seen in cleared brain section of pups (250 µm z-optical section of 2 mm thick section). (**E)** Close proximity of a GFP+ maternal cell to a blood vessel is shown. As it also expresses glial marker GFAP, it is likely to be an astrocyte associated with the blood vessel to contribute the blood brain barrier. (**F**) Orthogonal section of GFP+ cell detected at the top of the blood vessel (scale bars: 15 µm) (See also Movie S1).

Flow cytometry analysis showed that maternal cells can be transferred to the blood and brain of the pups via breast milk. To verify presence of these cells and identify their phenotype in the pups’ brains tissue clearing and immunocytochemistry were performed. For this, brain sections were double stained with primary antibodies (anti-GFP/anti-GFAP and anti-GFP/anti-NeuN). Triple positive cells (GFP^+^/anti-GFP^+^/anti-NeuN^+^ and GFP^+^/anti-GFP^+^/anti-GFAP^+^) were examined in whole coronal brain sections from Bregma +2 mm to −3,5 mm. These cells were widely distributed from the cerebral cortex to the different midbrain regions. We detected them in the deep cortical plate, dispersed among fiber tracts such as corpus callosum, in and around the hippocampal region, striatum, thalamus and hypothalamus. Throughout the brain we could not identify a specific brain region where GFP+ maternal cells were preferentially concentrated. The GFP signal was co-localized both with neuronal marker NeuN and glial protein GFAP immunoreactivity (Figs 2 and 3, see also Supplementary Figs S1 and S2 and Movies S1–S3).

### Detection of EGFP Copy Number in Brain of Pups by qPCR

C_T_ values for each sample were taken as average of each replicate, log copy numbers were calculated using the standard curve equation. After comparison from log copy number to copy number, presence of breast milk originating GFP+ cell in brain tissues of 1-week and 2-months old pups were determined quantitatively as shown in Table 1.

**Table 1:** Real-time PCR analysis of brain of sucklings.

DNA Sample	Average CT value	Log copy No/100 ng	Copy No/100 ng
No Template Control	—	—	—
WT	—	—	—
GFP+	23.02 ± 0.02	4.8 ± 0.004	53652
1-week old pups	34.74 ± 1.0	1.2 ± 0.3	197
2-months old pups	34.98 ± 1.2	1.1 ± 0.3	165

Genomic DNA was extracted from the brain tissue of pups and qPCR was performed. Serial dilutions of the pEGFP-N3 plasmid (1 pg/μl to1 ag/μl) were used to construct a standard curve in each assay. Plasmid standard curve linear equation: y = −3.235x +38.637; where y is CT EGFP (Cycle Threshold) and x is Log copy number. Average amplification efficiency = 99.8%. Average CT (cycle threshold) values from duplicate samples assayed in three separate experiments.

## Discussion

Breast milk has always attracted research, traditionally as an invaluable nutritional source and recently as a medium containing various biologically active compounds and living cells^14–16^. The relationship of the cells in breast milk with the baby was first demonstrated in 1983^17^ and subsequent studies on the subject often focused on their immune interactions^9,10,18,19^. By the demonstration of immunoreactivity for multipotent stem cell marker nestin in the BMCs^8^ possibility of more diverse functions became the subject of new investigations. It was shown that embryonic stem cell - like cells isolated from the breast milk can differentiate into cells of various tissues from 3 different germ layers^3,4^. Hassiotou *et al*. performed a detailed analysis of BMCs’ fate in wildtype pups breastfed by TdTomato expressing foster mothers and reported their presence in the blood, stomach, thymus, liver pancreas, spleen and brain in stem cell or differentiated forms^13^. In the current study, we confirm and extend some of these findings by demonstrating that stem cells in the breast milk not only pass to the suckling’s blood circulation but also home in on the brain tissue where they differentiate into both neurons and glial cells.

The first challenge for the BMCs is to survive the harsh conditions in the gastrointestinal tract and then to enter the blood circulation. However, the neonatal organisms have some compromises for both. First, digestive enzymes and gastric acid in the digestive system are weaker in newborns^20,21^. It was reported that BMCs collected from stomach of 2-week old mice following suckling had a viability of 80%^22^. Second, the permeability of the intestines to macromolecules and cells in the neonatal period is higher than that of a fully developed intestine^23^. Experiments with different animal models, including mouse, baboon and lamb, demonstrated that milk leukocytes can penetrate the intestinal wall by diapedesis and pass into the blood circulation^10,17,24–27^. It has also been reported that the permeability of the Peyer’s patches in the intestines is greater than in other intestinal regions. Indeed, maternal T lymphocytes from the breast milk were detected in this tissue^9^. Once they pass to portal circulation, the first destination of BMCs would be the liver^10,28,29^. Consistent with other reports^13^, we found quite a large number of maternal cells in the suckling’s blood even after two months, a finding emphasizing the significance of the phenomenon. Another standing question is how BMC’s cross the blood brain barrier (BBB). Activated leukocytes may pass through the vascular endothelium to the brain parenchyma by a process consisting of cell adhesion, diapedesis and cell migration^30^. Intravascularly delivered neural and mesenchymal stem cells have been reported to cross BBB and migrate into the brain tissue by various mechanisms^31–33^. Interestingly, according to a recent report, stem cells may use a peculiar type of extravasation, referred to as angiopellosis, in which the stem cell is relatively passive while vascular wall undergoes remodeling to extrude it^34^. Moreover, new born BBB may be more permeable^35,36^, though some oppose this idea^37^. Though this study does not directly address the issue, existing literature suggests that like others, stem cells in the breast milk may also have strategies to cross BBB.

The presence of cells expressing pluripotency stemness markers such as Oct4, SOX2, NANOG, SSEA4 and TRA-1-60 in the breast milk has been shown and they were successfully differentiated into various cell types including neurons in culture^4,12^. In this study, we detected BMCs in the suckling’s brains that had differentiated both into neuron and glial cell types expressing NeuN and GFAP, respectively. It is noteworthy that these cells were detectable in the brain as early as P7. This is in line with others’ finding that BMCs can pass to the suckling from the beginning of the breast-feeding period before the functional development of the digestive tract is completed^3,17^. The fact that these cells are still present in the brain 2 months after birth, as Hassiotou *et al*. also demonstrated this in multiple tissue^13^, implies that this is not a simple early-life epiphenomenon, though its significance is not clear. Harboring foreign cells from another individual is called microchimerism, which occurs during pregnancy through the placenta, and postnatally by blood transfusion or stem cell, bone marrow, organ and tissue transplantation^38–40^. What we report in this study can be defined as breastfeeding-induced maternal microchimerism and probably has commonality with trans-placental version. The latter has been known for long and shown to involve many organ and tissues from the bone marrow to the heart^28,29,41^ though no report on the brain involvement has been published so far, which adds to the novelty of our findings. Fetal cells can also pass through the placenta and lead to fetal microchimerism^42^, which is out of scope of this paper.

Maternal microchimerism is quite common in human and may occur more than one fifth of the population, persists for decades and involves multiple organs and tissues^43,44^. This study supports the idea that breastfeeding may be partially responsible for this high incidence^3^. Based on the experimental data and theoretical calculations, daily viable BMCs transfer to a breastfed human baby was estimated to be 4 × 10^6^ to 17 × 10^9^, among which there are substantial number of stem cells^3^. Biological significance of maternal microchimerism is rather complicated^29^. Breastfeeding-induced maternal microchimerism is thought to induce immune system maturation and tolerance to non-inherited maternal antigens, which may also explain how these foreign cells persist in the pup tissues without inducing immune response^28,45^. Indeed, transplanted organ recepients who were breastfed as infants have higher tolerance to tissues from their mothers^46–48^. It is reported that maternal cells may engraft into child’s organs and contribute to the host tissue function as in the case of maternal cells detected in child’s heart as differentiated cardiomyocytes^28,49^. What significant roles maternal cells in the newborn’s brain may play is an enigmatic question. It has been proposed that they may contribute to tissue maturation and support the host cells by secreting growth factors^12^. Some authors suggest that breastmilk may cause epigenetic changes in the infant through, for example, exosomal microRNA content and this may even lead to a type of kinship even among non-siblings breastfed by the same female^50–54^. Engrafted maternal cells may act as a permanent source of exosome – mediated secretions and keep affecting the host tissue epigenetically and/or chemically, even after the weaning.

Maternal microchimerism has been often associated with pathological states especially with autoimmune diseases, notably systemic sclerosis, systemic lupus erythematosus and neonatal lupus syndrome^55,56^. Elevated levels of circulating maternal cells in the offspring are associated with type 1 diabetes^57,58^. Potential involvement of chimeric brain cells with diseases, especially of autoimmune nature should be considered and investigated. On the other hand, some authors emphasize the potential of breastmilk to easily and non-invasively harvest stem cells for emerging cell replacement therapies for various genetic and acquired diseases and injuries^3,4,12^.

In conclusion, this study has firmly established that BMCs may lead to microchimerism in the brain of mouse sucklings, while spectrum of implications remains to be elucidated.

## Materials and Methods

### Experimental Groups

All animal experiments were reviewed and approved by the Animal Research Ethics Committee of Istanbul Medipol University. All procedures were conducted in conformity with institutional guidelines that are in compliance with European Economic Community Council Directive 86/609. C57BL/6-Tg(CAG-EGFP)1Osb/J mice were obtained from the Jackson Laboratory (ME, USA, #003291). This mouse line, with an enhanced GFP (EGFP) cDNA under the control of a chicken beta-actin promoter and cytomegalovirus enhancer, have widespread EGFP fluorescence, with the exception of erythrocytes and hair, referred to as GFP+. Animals were housed in temperature, water, and humidity-controlled cages that alternated between 12 h light and dark cycles.

The basic experimental paradigm of the study was to let GFP+ mice breastfeed wildtype C57BL/6 (WT) pups and to track GFP+ cells in the brain tissue of pups at different time points (Fig. 1A). For this purpose, two main groups were formed: (1) Newborn WT pups were nursed by GFP+ dams (n = 24, all WT pups were nursed by 6 GFP+ mice), (2) WT pups were nursed by WT dams as a negative control (n = 12). Brain and blood samples from fostered pups were collected at 1-week (n = 12) and 2-months (n = 12) of nursing for experimental assays. 6 mice in each group were used for flow cytometric and real time PCR analysis while 6 mice were used for immunohistochemical staining and microscopic evaluations. Flow cytometry and real time PCR analysis were performed immediately after the animals were sacrificed. For microscopic evaluations, mice were transcardially perfused by 10 ml PBS followed by 10 ml 4% paraformaldehyde (PFA). After perfusion, brain samples were dissected and split into two parts by coronally cutting into 2 mm pieces; one part was processed for tissue clearing method as described below, the other part was incubated in 20% sucrose-PBS until samples sank at the bottom of the tube, embedded in OCT compound (Tissue-Tek®) and stored at −80 °C until sectioning.

### Isolation of Cells from Bloodstream and Brain

Cells were isolated from blood and brain samples for flow cytometric analysis to identify GFP+ cells in the tissues.

Blood samples were lysed to remove erythrocytes. Briefly, 2 ml of BD Pharm Lyse ™ (BD Biosciences, 555899) was added to 200 μl of blood sample and incubated for 15 min at RT. Samples centrifuged at 200 g for 5 min and supernatant was discarded. then 2 ml PBS was added onto the pellet and centrifuged at 200 g for 5 min. After the supernatant was discarded, 500 μl PBS was added onto the pellet to obtain cells.

Brain tissues were divided into pieces with a thickness of 1 mm with a razor blade and taken into a Hibernate-A (ThermoFisher Scientific, A1247501) solution. For enzymatic digestion, they were incubated with 4 mg/ml papain (Sigma-Aldrich, P3125) for 1 h at 30 °C and then triturated. After centrifugation for 5 min at 500 G, the supernatant was discarded and cells were obtained in pellet. Obtained cells were fixed with BD Cytofix/Cytoperm (BD Biosciences, 554722).

### Flow Cytometry

Isolated cells were first treated with blocking solution (3% bovine serum albumin, 0.3% sodium azide, 1% goat serum, 0.1% Triton X-100) to block Fc receptors. They were then labeled with Alexa Fluor conjugated anti-GFP antibodies to confirm and enhance GFP signals. Subsequently, each sample was stained with DAPI or TO-PRO3 and then analyzed by flow cytometry. Flow cytometry was performed with BD Influx™ Cell Sorter (BD Biosciences). Analysis and absolute cell count were performed after acquiring at least 50,000 events. Doublet and multiplet discrimination were performed by using side scatter (SSC)-W vs. SSC-H and forward scatter (FSC)-W vs. FSC-H plots. Recorded data were analyzed using FlowJo (Tree Star) software.

#### Gating Strategy

The first orientating gate was selected using FSC vs. SSC plot to exclude particles and debris smaller than the cells were excluded from the analysis. Subsequently, DAPI or TO-PRO3 vs. FSC plots were used to select intact cells with nuclei. After that, respectively, GFP and anti-GFP vs. FSC plots were used to identify the cells which was transferred through breast milk of GFP+ foster mothers to WT pups. Identified cells were sorted in separate tubes from the samples for microscopic evaluation.

### DNA Extraction from Brain Tissue

Brain tissues of 1-week and 2-months old pups were collected and total genomic DNA were isolated using Qiagen DNeasy Tissue Kit (Qiagen, Valencia, CA, USA) by applying protocol provided by the manufacturer. DNA concentrations were determined using Qubit dsDNA HS Assay Kit (Thermo Scientific, Q32851).

### Real-Time PCR

Real-time PCR was performed to determine the copy number of GFP+ cells in DNA lysates isolated from brain tissues of the pups. To determine the exact copy number of GFP+ cells, standard curve changing between 2 to 2 × 10^5^ copy number/µl was constructed with serially diluted pEGFP-N3 plasmids which is a kind gift from Dr. Fahri Akbaş. For ng/µl to copy number/µl conversion of pEGFP-N3 plasmid, formula below is used as Joshi *et al*. reported^59^.

1 $$m=[n]\ast [\frac{1\,mol}{6.02\ast {10}^{23}}]\ast [\frac{660\,g}{mol}]=[n]\ast [1.096\ast {10}^{-21}\frac{g}{bp}]$$

Equation used for ng/µl to copy number/µl conversion (n: DNA length, bp: base pair, m: mass, average molecular weight of 1 mole dsDNA molecule: 660 g/mole).

For achieving high sensitivity to detect single copy number of genomic DNA, probe based real-time PCR was performed. Probe and primer sequences specific to C57BL/6-Tg(CAG-EGFP)1Osb/J mice strain were obtained from Jackson Laboratory and synthesized in Ella Biotech. Probe, forward and reverse primer sequences were 5′-Fam-TTC AAG TCC GCC ATG CCC GAA-Tamra-3′, 5′-AGT GCT TCA GCC GCT ACC-3′, 5′-GAA GAT GGT GCG CTC CTG-3′ respectively. Each PCR reaction was conducted as triplicate in 20 µl volume using Fast Probe Master Mix (Biotium, 31005) with Bio-Rad CFX Connect™ (Bio Rad, CA, USA). Non-template, negative and positive controls were included in all experiments. Transgene copy numbers in each DNA lysate were calculated from the measured C_T_ values using the copy number-C_T_ equation depicted from the standard curve.

### Immunohistochemistry

Brains were cut using a cryostat (Leica Biosystems, CM 1850) (coronal sections of 20 µm thickness) and mounted on positively charged glass slides (ThermoFisher Scientific, 10143352). Sections were fixed in 4% paraformaldehyde (PFA)/0.1 mol/L phosphate buffered saline (PBS) for 30 mins, washed and immersed for 1 h in blocking solution (0.1 mol/L PBS containing 0.3% Triton X-100 (PBS-T)/10% normal goat serum). Sections were incubated overnight (o/n) at 4 °C with Alexa Fluor 555-conjugated monoclonal mouse anti-NeuN (Merck Millipore, MAB377A5, 1:500), DyLight 550-conjugated monoclonal Mouse anti-GFAP (Novus Biologicals, NBP2-33184R, 1:1000) and Alexa Fluor 405-conjugated rabbit polyclonal anti-GFP (Novus Biologicals, NB600-310AF405, 1:100). After that, sections were washed with PBS, counterstained with TO-PRO^®^3 (ThermoFisher Scientific, T3605) and analyzed using a laser scanning confocal microscope (LSM 780, Carl Zeiss, Jena, Germany).

### Whole Tissue Immunohistochemical Staining and Clearing

Tissue clearing and immunohistochemically staining method were used for brain samples as described^60^. Briefly, dissected brains were coronally cut into 2 mm pieces. Brain slices were fixed in 4% PFA at 4 °C o/n with shaking and washed in PBS for 30 mins at room temperature (RT). Before the immunohistochemical staining, sections were pretreated with methanol/H_2_O series: 20%, 40%, 60%, 80%, 100%; 1 h each. After that, sections were incubated in 66% dichloromethane (Sigma Aldrich, 270997, DCM)/33% methanol at RT. Then, the samples were washed twice in 100% methanol at RT and chilled at 4 °C. Sections were bleached in chilled fresh 5% H_2_O_2_ in methanol o/n at 4 °C and rehydrated with methanol/H_2_O series: 80%, 60%, 40%, 20%, PBS; 1 h each at RT. After pretreatment, sections were incubated at 37 °C and permeabilized in PBS-T for one day and blocked in blocking solution for another day. Subsequently, they were incubated with Alexa Fluor 555 or DyLight 550 conjugated primary antibodies (anti-NeuN and anti-GFAP) for 2 days at 37 °C. Washed in PBS-T for 4–5 times until the next day and counter stained with TO-PRO^®^3.

After immunohistochemical staining, sections were cleared for further microscopic investigations. Sections were dehydrated in methanol/H_2_O series: 20%, 40%, 60%, 80%, 100%; 1 h each and incubated in 66% DCM/33% methanol for 3 h at RT. Then they were incubated in 100% DCM for 15 mins twice to wash methanol. Finally, sections were incubated in DiBenzyl Ether (Sigma Aldrich, 108014, DBE) until the sections were optically transparent. After the tissue clearing, brain samples directly imaged in laser scanning confocal microscope in a chamber filled with DBE.

### Statistics

The Graph Pad Prism 6 (GraphPad Software Inc., CA, USA) was used for statistical analysis. Student’s t test, one-way ANOVA and Pearson’s correlation analyses were used where appropriate.

## Electronic supplementary material

Supplementary Movie S1

Supplementary Movie S2

Supplementary Movie S3

Supplemental Information
