# Supplementary material for: Transfer and Integration of Breast Milk Stem Cells to the Brain of Suckling Pups
Source: Sci Rep. 2018 Sep 24;8:14289. doi: 10.1038/s41598-018-32715-5 (PMC6155265; doi:10.1038/s41598-018-32715-5)
Supplement: Supplementary file 4 — Supplemental Information [file 41598_2018_32715_MOESM4_ESM.pdf]

SUPPLEMENTARY INFORMATION

Transfer and Integration of Breast Milk Stem Cells to the Brain of Suckling Pups

Mehmet Şerif Aydın[1], Esra Nur Yiğit[1], Emre Vatandaşlar[1], Ender Erdoğan[2] and Gürkan Öztürk[1,3,*]

		The percentage of GFP+ cells in total cells	
**Groups**	**Number of Mice**	**Brain**	**Blood**
**1 week old**	**#1**	0.42	6.22
	**#2**	0.14	4.93
	**#3**	0.11	1.40
	**#4**	0.18	6.21
	**#5**	0.04	7.10
	**#6**	0.03	5.25
**2 months old**	**#1**	0.02	5.88
	**#2**	0.50	5.20
	**#3**	0.51	4.94
	**#4**	0.10	1.55
	**#5**	0.11	6.20
	**#6**	0.04	5.02

Supplementary Table S1: The percentage of GFP+ cells in total cells detected by flow cytometry in the 1-week old (n=6) and 2-months old (n=6) groups.

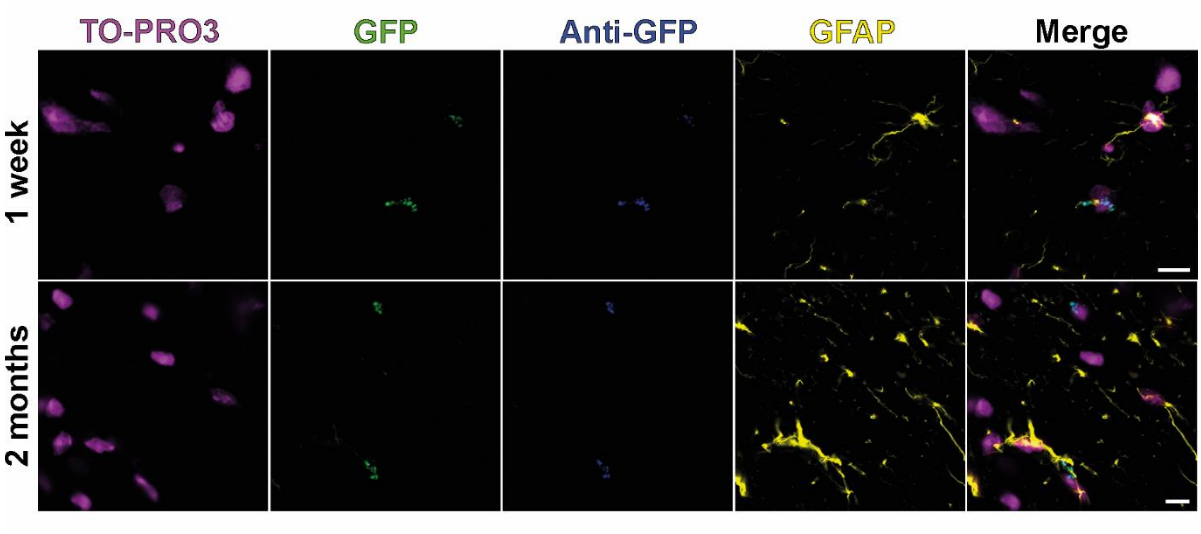

Supplementary Figure S1: GFP+ cells differentiated to astrocytes (GFAP+) and unspecified type of cells (GFAP-/NeuN-) were detected in the brain after 1 week and 2 months of nursing (scale bars: 10μm).

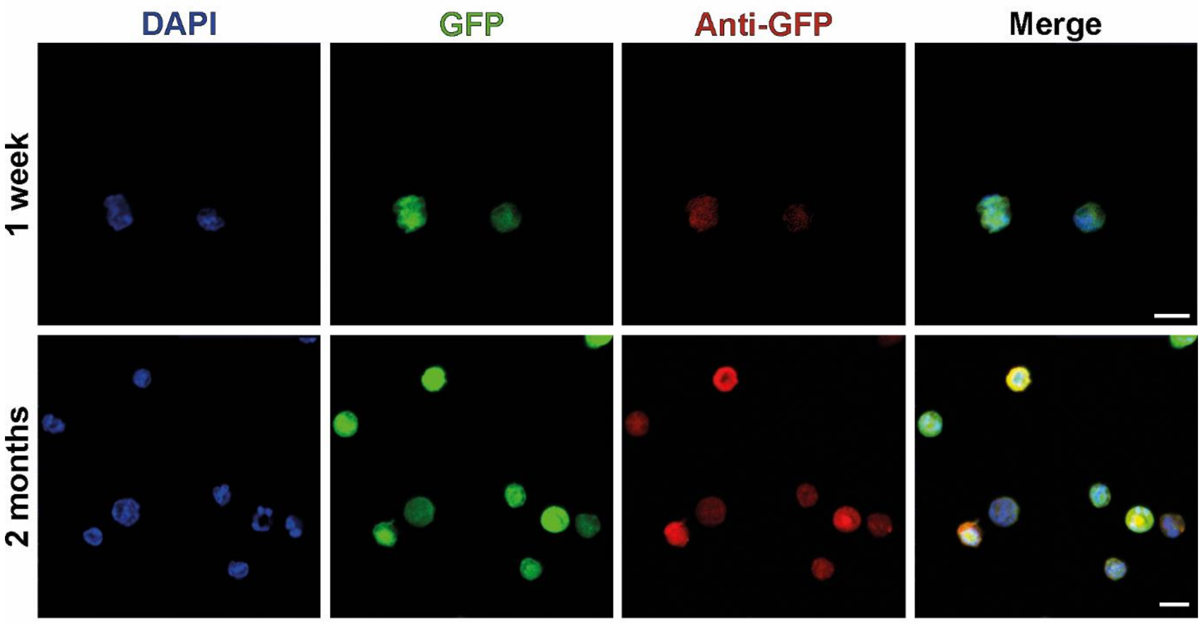

Supplementary Figure S2: GFP+ cells detected in the 1-week and 2-months old suckling's bone marrow. Bone marrows were flushed out from mouse femur bone by syringe and processed in the same way as blood samples for flow cytometric analysis. Anti-GFP labeled GFP+ cells were sorted and verified by laser scanning confocal microscope. (scale bars: 10μm).

Supplementary Movie S1: 3-dimensional microscopic view of the cleared suckling's brain.

Supplementary Movie S2: 3-dimensional microscopic view of the GFAP+ (yellow), GFP+ (green) and anti-GFP+ (blue) cell in the brain of 1week old suckling pup.

Supplementary Movie S3: 3-dimensional microscopic view of the GFAP+ (yellow), GFP+ (green) and anti-GFP+ (blue) cell in the brain of 2 months old suckling pup.
